# Reorganizing Polymer Chains with Cyclodextrins

**DOI:** 10.3390/polym9120673

**Published:** 2017-12-04

**Authors:** Alper Gurarslan, Abhay Joijode, Jialong Shen, Ganesh Narayanan, Gerry J. Antony, Shanshan Li, Yavuz Caydamli, Alan E. Tonelli

**Affiliations:** Fiber & Polymer Science Program, College of Textiles, North Carolina State University, Raleigh, NC 27606-8301, USA; gurarslan@itu.edu.tr (A.G.); asjoijod@ncsu.edu (A.J.); jshen3@ncsu.edu (J.S.); gnaraya@ncsu.edu (G.N.); gajohn@ncsu.edu (G.J.A.); sli31@ncsu.edu (S.L.); ycaydam@ncsu.edu (Y.C.)

**Keywords:** polymers, cyclodextrins, inclusion compounds, coalescence

## Abstract

During the past several years, we have been utilizing cyclodextrins (CDs) to nanostructure polymers into bulk samples whose chain organizations, properties, and behaviors are quite distinct from neat bulk samples obtained from their solutions and melts. We first form non-covalently bonded inclusion complexes (ICs) between CD hosts and guest polymers, where the guest chains are highly extended and separately occupy the narrow channels (~0.5–1.0 nm in diameter) formed by the columnar arrangement of CDs in the IC crystals. Careful removal of the host crystalline CD lattice from the polymer-CD-IC crystals leads to coalescence of the guest polymer chains into bulk samples, which we have repeatedly observed to behave distinctly from those produced from their solutions or melts. While amorphous polymers coalesced from their CD-ICs evidence significantly higher glass-transition temperatures, *T*_g_s, polymers that crystallize generally show higher melting and crystallization temperatures (*T*_m_s, *T*_c_s), and some-times different crystalline polymorphs, when they are coalesced from their CD-ICs. Formation of CD-ICs containing two or more guest homopolymers or with block copolymers can result in coalesced samples which exhibit intimate mixing between their common homopolymer chains or between the blocks of the copolymer. On a more practically relevant level, the distinct organizations and behaviors observed for polymer samples coalesced from their CD-ICs are found to be stable to extended annealing at temperatures above their *T*_g_s and *T*_m_s. We believe this is a consequence of the structural organization of the crystalline polymer-CD-ICs, where the guest polymer chains included in host-IC crystals are separated and confined to occupy the narrow channels formed by the host CDs during IC crystallization. Substantial degrees of the extended and un-entangled natures of the IC-included chains are apparently retained upon coalescence, and are resistant to high temperature annealing. Following the careful removal of the host CD lattice from each randomly oriented IC crystal, the guest polymer chains now occupying a much-reduced volume may be somewhat “nematically” oriented, resulting in a collection of randomly oriented “nematic” regions of largely extended and un-entangled coalesced guest chains. The suggested randomly oriented nematic domain organization of guest polymers might explain why even at high temperatures their transformation to randomly-coiling, interpenetrated, and entangled melts might be difficult. In addition, the behaviors and uses of polymers coalesced from their CD-ICs are briefly described and summarized here, and we attempted to draw conclusions from and relationships between their behaviors and the unique chain organizations and conformations achieved upon coalescence.

## 1. Introduction

Unlike atomic and small molecule solids, the behaviors and properties of materials made from polymers can be significantly altered during their processing, because they are closely related to the organizations, structures, and morphologies of their constituent chains. The conformations and arrangements of their inherently flexible long chains are amenable to modifications through processing, so materials made from the same polymer can behave very distinctly when different means are used to process them. One need only compare the behaviors of garbage bags and gel-spun Spectra^®^ fibers both made from poly(ethylene) (PE). Even though the “same” polymer is used in both applications, the highly oriented and crystalline PE chains in Spectra PE produce extremely strong fibers and may be fabricated into lightweight armor, while molded articles, such as melt-blown PE garbage bags, with randomly-coiled and entangled PE chains, are not nearly as strong, but have a much greater elasticity. The widely different means used to process PE Spectra fibers and PE garbage bags produce widely different organizations, structures, and morphologies of their polymer chains and resultant properties.

**Figure 1:**
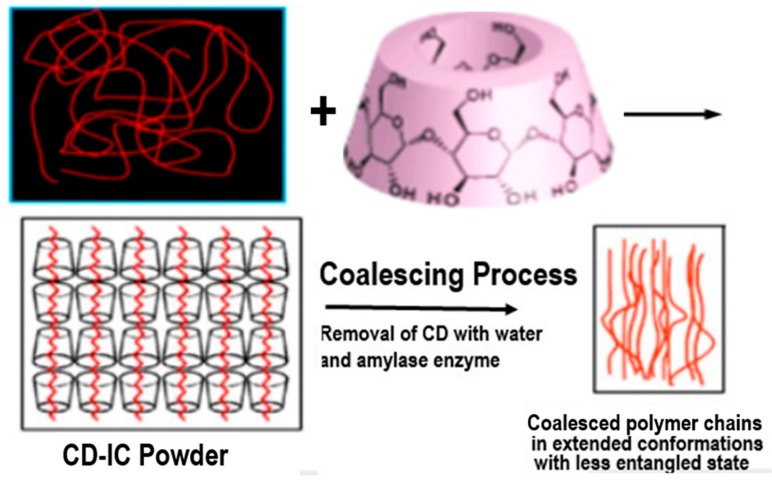
Formation of and coalescence of a polymer sample from its crystalline cyclodextrin complex. Figure adapted with permission from Reference [2], Copyright 2009 Springer-Verlag London 2009.

Here we describe a means for nano-processing polymers into solids exhibiting unique properties. First, we form non-covalently bonded inclusion compounds (ICs) between guest polymers and host cyclodextrins (CDs) [1,2]. The minimum cross-sectional areas of guest polymers adopting highly extended conformations determines which CD can include them. This is followed by coalescing the guest polymer chains into a bulk solid sample by carefully removing the host CDs. In Figure 1, CDs are shown to form ICs with guest polymers, which are included and reside in the very narrow CD-IC channels (~0.5–1.0 nm in diameter) formed by their crystalline host lattice [3]. There, the guest chains are stretched to high extension and isolated from other chains. It was hoped that, through the careful removal of the crystalline host CD lattice, the resulting coalesced polymer chains (c-polymers) would retain a significant degree of their included extended and un-entangled natures, so they would be organized in a manner quite different from samples processed from their solutions or melts, where they randomly coil and entangle. As we will now demonstrate, this has indeed been found to be the case [2,3,4,5,6,7,8,9,10,11,12,13,14,15,16,17,18,19,20,21,22,23,24,25,26,27,28,29,30,31,32,33,34,35,36,37,38,39,40,41,42,43,44,45,46,47,48,49,50,51,52,53,54,55,56,57,58,59,60,61,62,63,64,65,66,67,68,69,70,71,72,73]. Their behaviors and properties differ significantly from, and are improved with respect to those of ordinarily processed samples.

Some of the results presented here were previously summarized [74] and their experimental details can be found in the original papers [2,3,4,5,6,7,8,9,10,11,12,13,14,15,16,17,18,19,20,21,22,23,24,25,26,27,28,29,30,31,32,33,34,35,36,37,38,39,40,41,42,43,44,45,46,47,48,49,50,51,52,53,54,55,56,57,58,59,60,61,62,63,64,65,66,67,68,69,70,71,72,73].

## 2. Polymers Reorganized through Complexation with and Coalescence from Their CD-ICs

### 2.1. Amorphous Polymers

**Figure 2:**
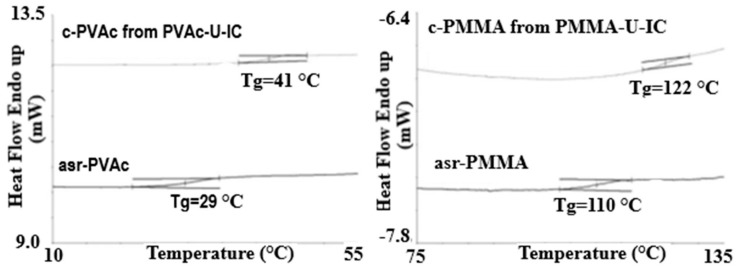
Differential Scanning Calorimetry (DSC) observed glass transitions in amorphous polymers as-received (asr) and coalesced (c) from their U-ICs. Figure adapted with permission from Reference [70], Copyright 2013 Wiley Periodicals, Inc.

**Figure 3:**
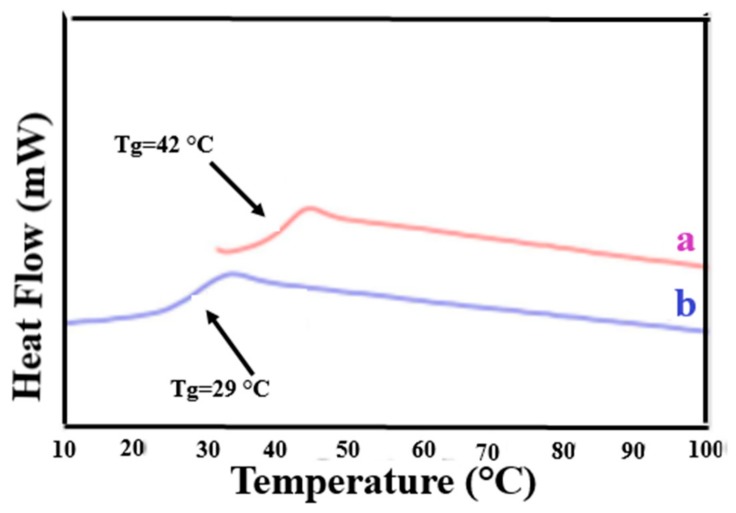
DSC thermograms of the second heating scan of: (**a**) c-PVAc from its γ-CD-IC; and (**b**) asr-PVAc. Figure adapted with permission from Reference [43]. Copyright 2005 Elsevier Ltd.

Figure 2 presents the 2nd heating DSC scans of as-received (asr) and coalesced (c) samples of the amorphous polymers atactic poly (vinyl acetate) (PVAc) and atactic poly(methyl methacrylate) (PMMA) [70]. These coalesced samples were in fact obtained from their urea (U)-ICs, not their CD-ICs. However, we have demonstrated [43,64,67,70] that c-polymers obtained from their CD- and U-ICs behave in virtually identical manners. Compare the *T*_g_ of c-PVAc obtained from its γ-CD-IC [43] in Figure 3 to that of the c-PVAc obtained from its U-IC shown in Figure 2. Note, in both cases, the *T*_g_ of the c-PVAc is raised more than 10 °C above the *T*_g_ of asr-PVAc. The *T*_g_ observed for c-PMMA is similarly elevated above that of asr-PMMA. It should be noted that in their CD- and U-ICs the PMMA and PVAc are isolated from each other and consequently do not experience a *T*_g_ or show any other change in heat capacity until the IC samples degrade above 250 °C.

Table 1 summarizes density measurements performed on asr- and c-PVAc samples both below and above their glass-transition temperatures. Note that PVAc coalesced from either its γ-CD- or U-IC is denser than asr-PVAc, both below and above their *T*_g_s. This increase in density is likely also due to the more extended conformations and closer packing of chains in c-PVAcs suggested in Figure 1.

**Figure 4:**
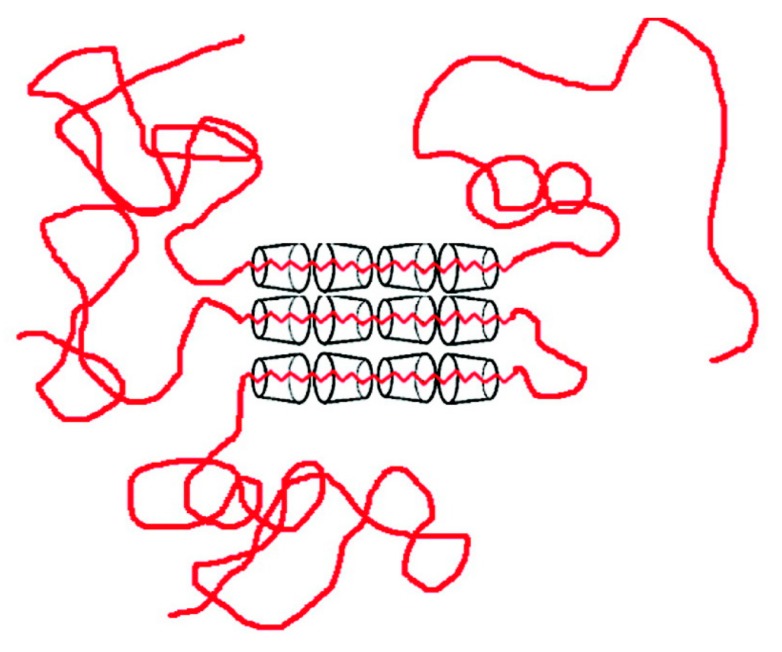
Schematic depiction of a (n-s)-polymer-CD-IC sample. Adapted with permission from Reference [57] Mohan, A.; Joyner, X.; Kotek, R.; Tonelli, A.E. *Macromolecules* **2009**, *42*, 8983–8991. Copyright 2009 American Chemical Society.

**Figure 5:**
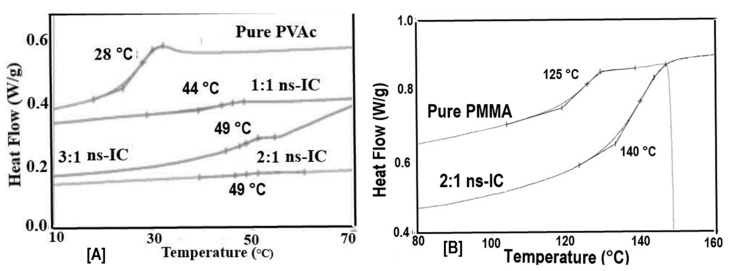
Heating DSC scans of (**a**) asr-PVAc and (**b**) asr-PMMA and their (n-s)-c-CD-ICs. The 3:1 and 2:1 ratios signify the amounts of PVAc and PMMA chains in excess of their stoichiometric amounts. Figure adapted with permission from Reference [70], Copyright 2013 Wiley Periodicals, Inc.

If we use an excess of guest polymer when forming an IC with host CDs, a non-stoichiometric (n-s)-polymer-CD-IC is formed, where portions of the guest chains are un-included and dangle outside the crystalline host CD lattice (see Figure 4). As evidenced in Figure 5, the *T*_g_s of the un-included portions of PVAc and PMMA chains in their (n-s)-γ-CD-ICs are significantly elevated above their neat bulk asr-samples. In fact, even though they were never included in the narrow channels created by the γ-CD-IC crystalline lattice, the un-included chains in (n-s)-CD-ICs may have *T*_g_s even higher than those of samples completely coalesced from either their stoichiometric- or (n-s)-CD-ICs. This has been suggested to be a result of the high density and high extension of the brush-like un-included chain portions emanating from the CD crystal surfaces of (n-s)-CD-ICs (see Figure 4 and subsequently Figure 17) [57,61].

**Figure 6:**
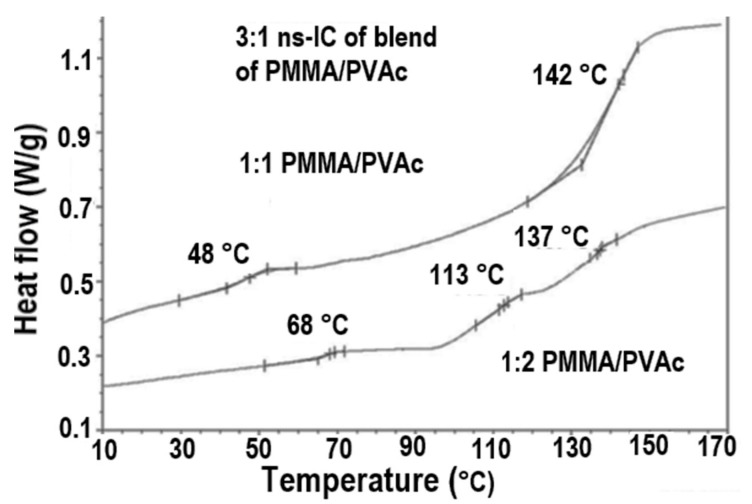
Heating DSC scans of (n-s)-common-PVAc/PMMA-c-CD-ICs. Figure adapted with permission from Reference [70], Copyright 2013 Wiley Periodicals, Inc.

We may also begin to blend and mix the un-included portions of the PVAc and PMMA chains by forming common (n-s)-γ-CD-ICs containing both polymers. The *T*_g_s of the un-included portions of PVAc and PMMA chains in their common (n-s)-γ-CD-ICs are presented in Figure 6. There, it is very clear, by comparison to the *T*_g_s observed in Figure 5, that the common 3:1 (n-s)-γ-CD-IC made using a 1:1 PVAc: PMMA molar ratio results in very little mixing between the un-included portions of the PVAc and PMMA chains. On the other hand, when a 2:1 molar ratio of PVAc: PMMA was used to make their common (n-s)-γ-CD-IC, substantial mixing of the un-included portions of PVAc and PMMA chains was generated, because the *T*_g_s of segregated un-included PVAc and PMMA chains are not observed. Instead, three distinct *T*_g_s are observed, and they may be attributed to PVAc-rich and PMMA-rich phases, and a well-mixed PVAc/PMMA phase.

**Figure 7:**
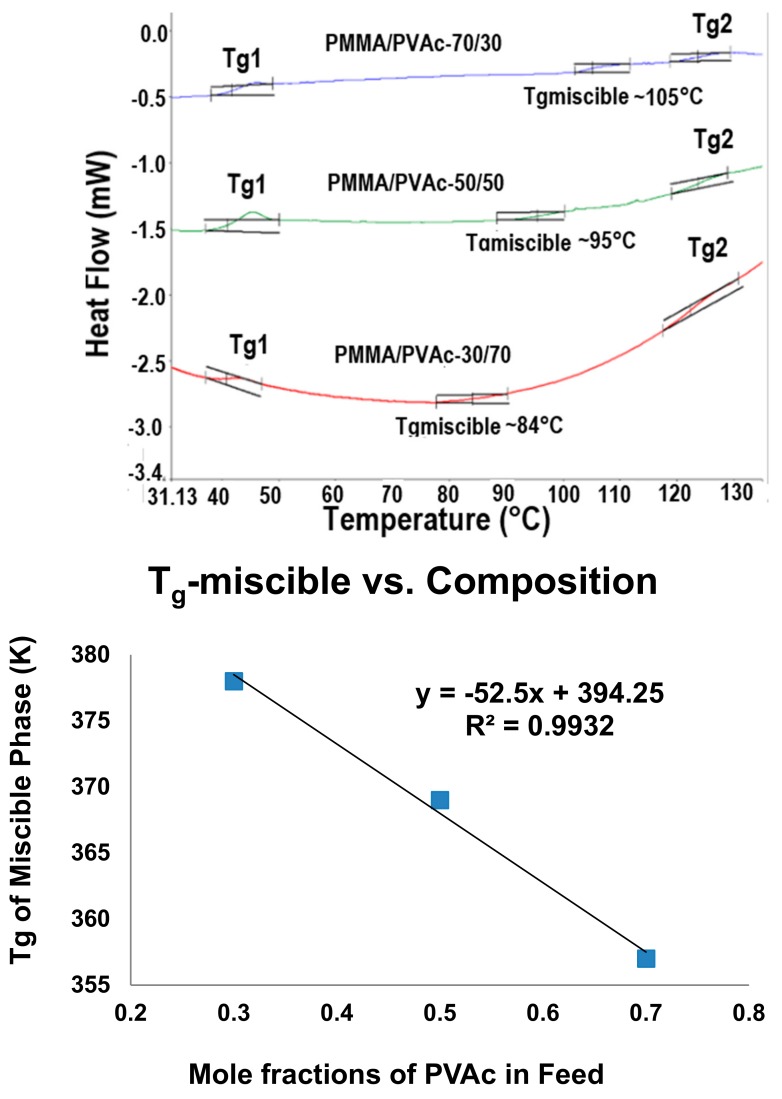
The DSC observed *T*_g_s observed from PVAc/PMMA blends achieved upon coalescence of their common stoichiometric U-ICs (**top**); and plotted versus their composition (**bottom**) [72].

The blending of inherently immiscible amorphous polymers can also be achieved through formation of and coalescence from their fully covered chain included common stoichiometric ICs, as illustrated in Figure 7 for PVAc/PMMA blends coalesced from their U-ICs [72]. They are not completely mixed and appear to possess three phases: PVAc and PMMA rich phases with *T*_g_s similar to those of their neat coalesced samples and well-mixed samples with intermediate *T*_g_s. It is interesting to notice that the *T*_g_s of the well mixed phases in their coalesced blends seem to be proportional to their molar compositions.

### 2.2. Semi-Crystalline Polymers

**Figure 8:**
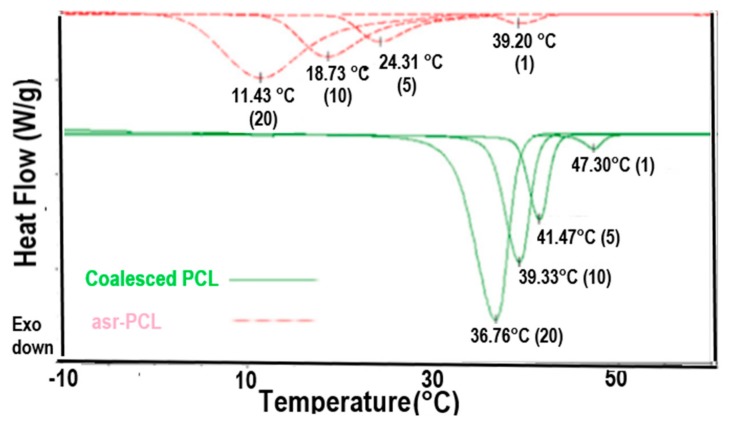
Melt-crystallization curves of asr-s and c-PCL observed at 20, 10, 5 and 1 °C/min cooling rates by DSC. Figure adapted with permission from Reference [64], Copyright 2011 Elsevier Ltd.

**Figure 9:**
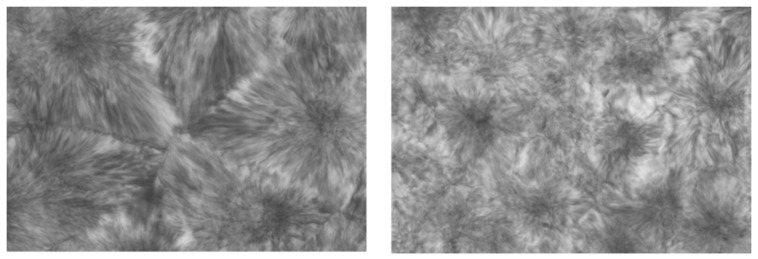
Optical microscopy images (500×), crossed polarizers, ¼ λ plate) of: melt pressed films of asr-PCL (**left**); and c-PCL obtained from its U-IC (**right**). Unpublished research from Prof. Tonelli’s research group.

Polymers that can crystallize also form ICs with CD and U hosts. Figure 8 presents the DSC cooling scans observed at different cooling rates for melts of asr-poly(ε-caprolactone) (asr-PCL) and c-PCL, the latter obtained from its α-CD-IC. Though both PCL samples crystallize at decreasingly lower temperatures the faster their melts are cooled, the c-PCL melt crystallizes at significantly higher temperatures than the asr-PCL melt regardless of the cooling rate. asr-PCL and c-PCL, with the latter obtained from its urea-inclusion compound (U-IC), crystallize from their melts upon cooling at −20 °C/min, respectively, at 11.7 and 33.3 °C [67], while as seen below in Figure 8, the same PCL coalesced from its IC formed with host α-CD crystallized at *T*_c_ = 36.8 °C from the melt upon cooling at −20 °C/min [64]. In addition, crystalline exotherms observed on cooling c-PCL melts are substantially narrower than those shown by asr-PCL. Upon cooling from the melt, not only does the c-PCL begin to crystallize sooner than asr-PCL, but its crystallization ceases much more abruptly than that of asr-PCL. Both features cause the semi-crystalline morphology c-PCL to be finer and more uniform, i.e., more homogeneous, as can be seen in Figure 9, and lead to improved mechanical and other properties.

**Figure 10:**
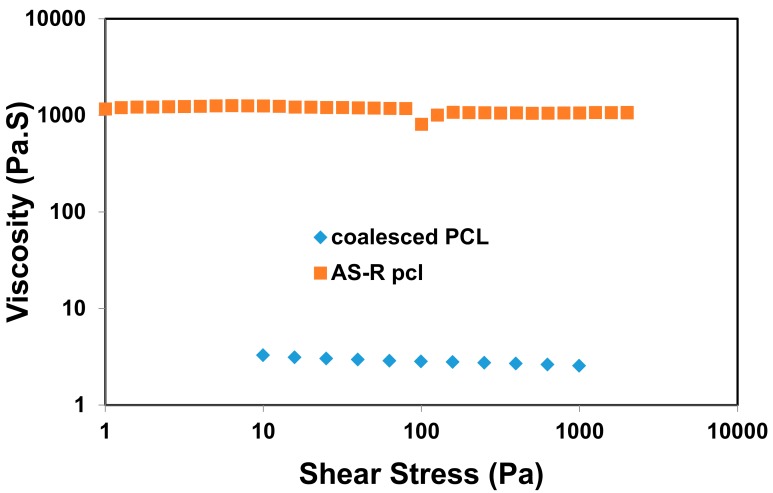
Comparison of frequency sweep rheology of as-received and coalesced PCL melts at *T* = 100 °C. Unpublished research from Prof. Tonelli’s research group.

In Figure 10, the melt rheology of asr- and c-PCL samples measured at *T* = 100 °C are compared and reveal a dramatic difference. Notice the ~100-fold decrease in the viscosity of the c-PCL melt, possibly the result of more extended less entangled chains, which also lead to more facile crystallization, as well as faster flow.

**Figure 11:**
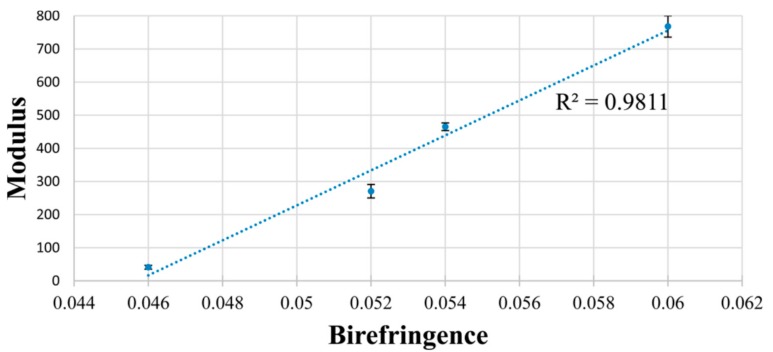
Correlation between modulus and birefringence of the four PCL fiber samples in Table 1. Error bars represent standard error. Reprinted (adapted) with permission from Gurarslan, A.; Caydamli, Y.; Shen, J.; Tse, S.; Yetukuri, M.; Tonelli, A.E. *Biomacromolecules* **2015**, *16*, 890–893 (Reference [73]). Copyright 2015 American Chemical Society.

Increased amounts of c-PCL were obtained from their U-ICs and were sufficient to permit melt-spinning of single filament fibers. These c-PCL fibers were tested mechanically and thermally before and after drawing, and their results were compared to fibers melt-spun from asr-PCL. Table 2 shows clearly that both before and after drawing the fibers obtained from c-PCL are superior in performance to the asr-PCL fibers. Figure 11 shows the strong correlation between the moduli of the undrawn and drawn c-PCL and asr-PCL fibers and their birefringence, which serves as a measure of the chain orientation in each fiber.

**Figure 12:**
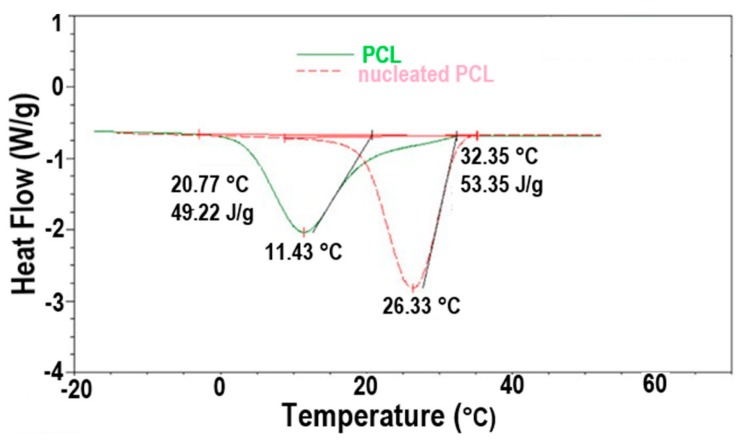
DSC −20 °C/min cooling scans of molten asr-PCL with and without 2.5 wt % c-PCL. Figure adapted with permission from Reference [64], Copyright 2011 Elsevier Ltd.

The improved crystallizability of c-polymers recommends their use as self-nucleants for asr-samples of the same polymer, as demonstrated for c-PCL in Figure 12. The DSC cooling scans of c-PCL and an asr-PCL sample to which 2.5 wt % of c-PCL has been added (nuc-PCL) are shown there. The self-nucleated PCL sample clearly exhibits an enhanced crystallizability and a finer more uniform morphology both produced by the higher temperature and narrower range of crystallization of the added c-PCL self-nucleant.

In Table 3, the densities and CO_2_ permeabilities of melt-pressed asr- and nuc-poly(ethylene terephthalate) (PET) films are presented [69]. The nuc-PET film was obtained by melt-pressing a physical mixture of 95 wt % asr-PET and 5 wt % c-PET. DSC observations of both largely amorphous melt-quenched PET films indicated similar crystallinities of ~10%. Clearly the nuc-PET film is denser than the asr-PET film and is markedly less permeable to CO_2_ even after both films were quenched from their melts into ice water. Both observations are consistent with the suggested higher orientation and more extended conformations of PET chains in the self-nucleated film, which likely increase their ordering and packing in the predominant amorphous domains.

**Figure 13:**
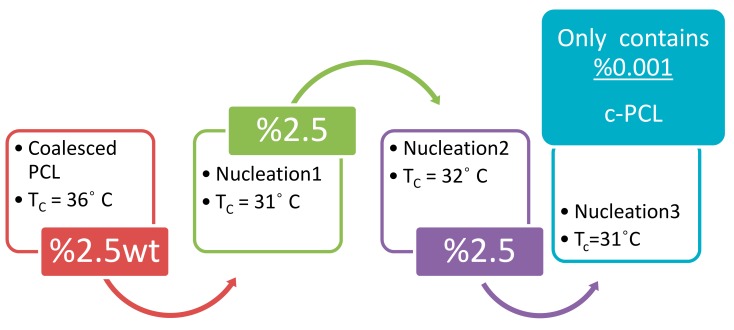
Sequential “Stealth” nucleation of molten asr-PCL (*T*_c_ = 11 °C) with c- and subsequently nuc-PCLs observed during −20 °C/min DSC cooling scans. Adapted with permission from Gurarslan, A.; Tonelli, A.E. ACS 2012, Spring 2012 National Meeting, Anaheim, CA (Reference [68]). Copyright 2012 American Chemical Society.

**Figure 14:**
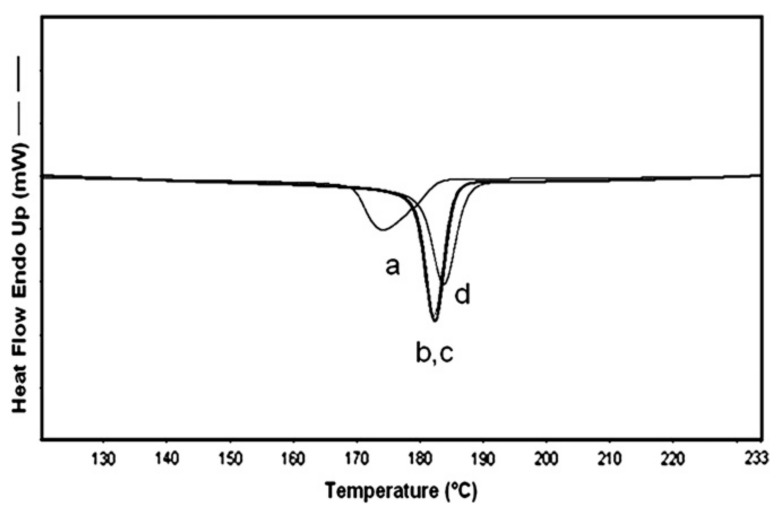
DSC cooling scans for (**a**) neat N-6 sample (*M*_W_ = 600,000 g/mol), with 2 wt % of (**b**) N-6 coalesced from a stoichiometric N6-α-CD-IC, (**c**) N-6 coalesced from a 3:1 (n-s)-N6-α-CD-IC, and (**d**) N-6 with 2 wt % talc. Adapted with permission from Reference [57] Mohan, A.; Joyner, X.; Kotek, R.; Tonelli, A.E. *Macromolecules* **2009**, *42*, 8983–8991. Copyright 2009 American Chemical Society.

Another advantageous feature of using c-polymers as self-nucleants is illustrated in Figure 13, where it can be seen that asr-PCL nucleated with c-PCL can itself be repeatedly used to nucleate additional asr-PCL. This means that very little c-polymer need be produced in order to serve as a self-nucleant for improving the morphologies and the resultant properties of semi-crystalline polymer materials. Additionally, using c-polymers as self-nucleants means the polymer materials they nucleate do not contain any foreign material, making them “Stealth” nucleants that are readily recyclable, and appropriate for medical applications (see Figure 14 for example).

## 3. Thermal Stability of the Chain Organization in Coalesced Polymer Samples

From DSC and density observations it is clear that c-PVAc is organized differently and behaves distinctly from asr-PVAc. Therefore, we conducted annealing studies to estimate the time taken for c-PVAc to revert back from its presumed extended un-entangled chain coalesced morphology to the randomly-coiling entangled chain morphology of asr-PVAc (see Figure 1). The *T*_g_s observed for asr-PVAc before and after annealing at 70 °C for 14 days were 29.1 and 29.4 °C, respectively, i.e., essentially the same. The *T*_g_s observed for c-PVAc obtained from its γ-CD after annealing at 70 °C for different times are presented in Table 4. They remain around 41 °C, so c-PVAc chains apparently remain largely extended and un-entangled even after annealing above their *T*_g_, without apparently returning to entangled random coils as in the asr-PVAc. It should also be noted that single concentration (0.2 g/dL) viscometer flow times for asr- and c-PVAcs measured in dioxane before and after annealing were both ~400 s, suggesting that long-time annealing above *T*_g_ (at 70 °C) had not caused any PVAc degradation.

**Figure 15:**
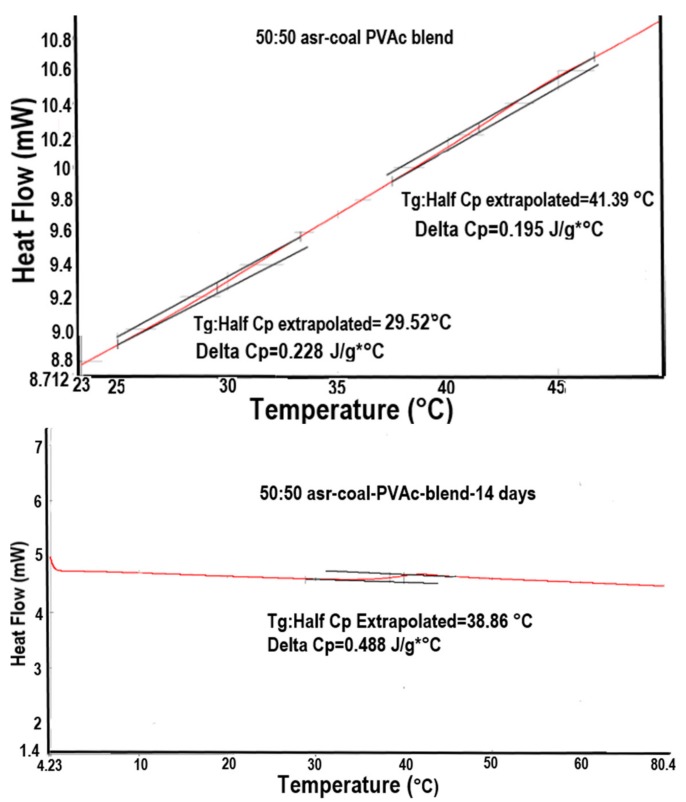
DSC scans of 50/50 physical mixture of asr-PVAc/c-PVAc: before (**top**); and after (**bottom**) annealing for 14 days above *T*_g_ at 70 °C. Adapted with permission from Reference [72]. Copyright Joijode et al. North Carolina State University 2014.

Even more intriguing are the tentative observations seen in Figure 15 [72], where DSC heating scans of a 50:50 physical mixture of asr-PVAc:c-PVAc recorded before and after long-time annealing above *T*_g_ are presented. While the initial heating scan reveals distinct *T*_g_s for phase separated asr- and c-PVAcs, after annealing for 14 days, apparently the 50/50 asr-PVAC/c-PVAC mixture is now a well-mixed blend, because a single *T*_g_ between those of asr-PVAc (29 °C) and c-PVAc (41 °C), but much closer to that of c-PVAc, is observed around 38 °C. After four weeks of annealing above *T*_g_ at 70 °C, the 50/50 asr-PVAc/c-PVAc blend has a *T*_g_ (DSC not shown) and density (Table 5) nearly identical to that of neat c-PVAc (from its γ-CD-IC).

The observation of a single *T*_g_ for the 50/50 asr-PVAc/c-PVAc (γ-CD) physical mixture annealed a long time above their *T*_g_s into a well-mixed blend is not surprising. However, the observation that the single *T*_g_ observed for their apparently well-mixed blend is close to that of c-PVAc, rather than asr-PVAc, is surprising. In other words, the blend is likely not characterized by completely entangled randomly-coiling PVAc chains, but rather by an organization of PVAc chains more similar to that of the initially coalesced neat c-PVAc (γ-CD) (see Figure 1).

Similarly, when semi-crystalline asr- and c-PCL (obtained from its α-CD-IC) samples were held in the melt for 1 week, 2 weeks, and 1 month at 90 °C, and subsequently observed by DSC, the following melt crystallization temperatures were observed at a −20 °C/min cooling rate: 35.6 (0), 33.4 (1 week), 32.6 (2 weeks), and 31.1 °C (1 month) for c-PCL and 11.4 (0) and 15 °C (2 weeks) for melt-annealed asr-PCL. In addition, viscometer flow times of 1 g/dL chloroform solutions of c-PCL before and after long term melt annealing were closely similar, indicating little if any thermal degradation had occurred. PCL coalesced from its U-IC before and after melt-annealing for 2–4 weeks were similarly crystallized at 33.3 (0), 30 (2 weeks), and 31.1 C (4 weeks) when cooled at −20 °C/min.

These results prompt the question: “What are the conformations and organization of the polymer chains in their c-samples, and why are they so resistant to long-time high temperature annealing?”

**Figure 16:**
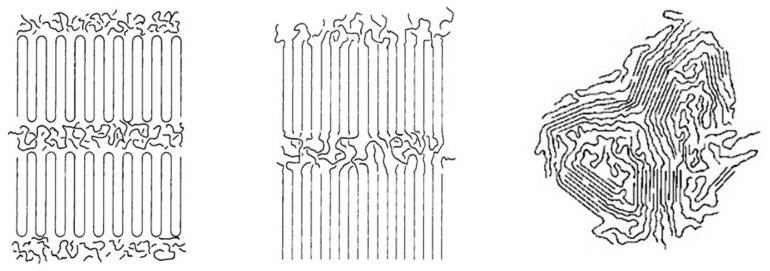
Idealized comparison of: regularly-folded single-chain lamellar crystals (**left**); irregularly-folded multi-chain “switchboard” lamellar crystals (**center**); and “fringed-micelle” semi-crystalline morphology (**right**). Adapted with permission from Reference [76]. Gurarslan, A.; Joijode, A.S.; Tonelli, A.E. *J. Polym. Sci. Part B Polym. Phys.* **2012**, *50*, 813–820. Copyright Wiley 2012.

Rastogi et al. [75] recently observed that slow and carefully controlled melting of ultra-high molecular weight, metallocene catalyzed, single-chain crystalline polyethylenes (UHMW-PEs), as illustrated in the drawing at the left in Figure 16, can produce heterogeneous melts, with more and less entangled regions. They observed that it took more than a day of heating at 180 °C to produce a fully entangled melt of randomly-coiling PE chains. On the other hand, melts of those polymers initially coalesced from their CD-IC crystals, such as those reported here for c-PVAc, with presumably extended and largely un-entangled chains, apparently take much longer to return to their randomly-coiling entangled melt state.

Quoting Rastogi et al. [75], “crystals composed of single chains are feasible, where the chains are fully separated from each other. If such separation can be maintained in the melt a new melt state can be formed. Here we show that through slow and carefully controlled melting such polymer crystals form a heterogeneous melt with more entangled regions, where the chains are mixed, and less entangled ones, composed of individually separated chains. Chain reptation, required for the homogenization of the entanglement distribution, is found to be considerably hindered. The long-lived heterogeneous melt shows decreased melt viscosity and provides enhanced drawability on crystallization.”

In contrast, the usual morphology of semi-crystalline polymers with intimately mixed chains, as illustrated by the central drawing in Figure 16, is rapidly converted upon heating into a homogeneous entangled melt of randomly-coiling chains. Chain reptation is required to produce homogeneous melts from both semi-crystalline morphologies. However, in the case of the slow heating of metallocene-catalyzed UHMW-PE nascent single-chain crystals, the initial rapid entanglements formed between portions of PE chains melting and detaching from the ends of different single-crystals serve to retard the further entanglement of the central portions of the PE chains, leading to a heterogeneous melt whose ultimate homogenization is thus greatly retarded [77].

**Figure 17:**
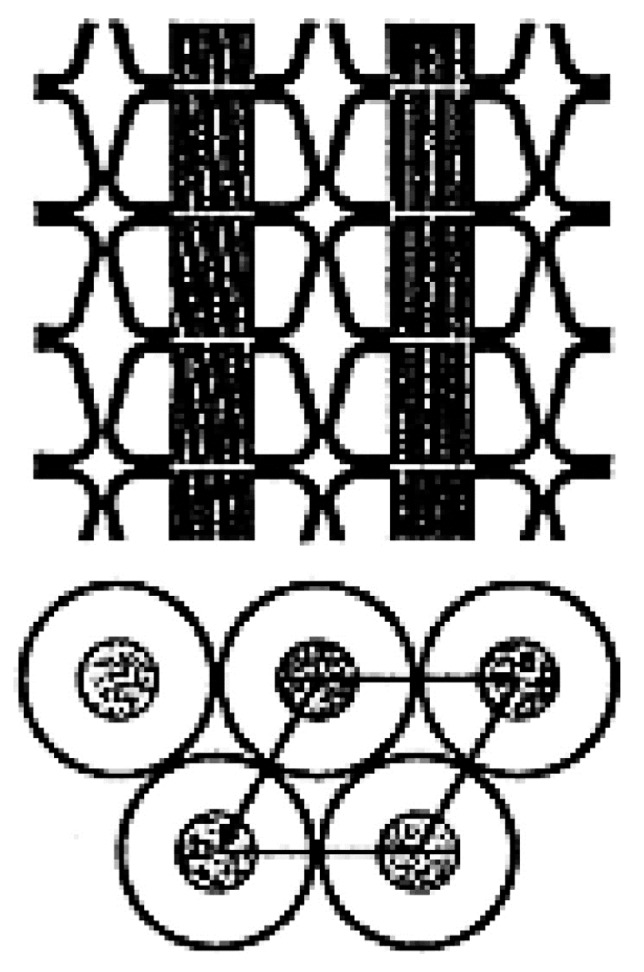
Schematic representation of a channel structure polymer-CD-IC. Adapted with permission from Reference [57] Mohan, A.; Joyner, X.; Kotek, R.; Tonelli, A.E. *Macromolecules* **2009**, *42*, 8983–8991. Copyright 2009 American Chemical Society.

**Figure 18:**
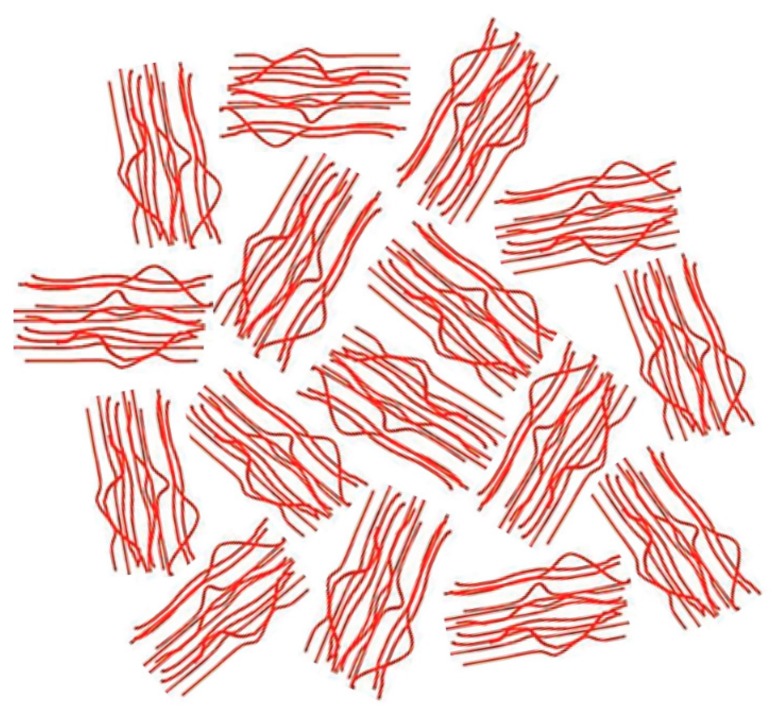
2-D representation of a polymer sample coalesced from its CD-IC. Unpublished work from Prof. Tonelli’s research group.

Because the coalescence process begins with a polycrystalline powder sample of the polymer-IC, with individual crystalline grains that are randomly oriented. Upon careful removal of the CD or U hosts, the volume of each resultant coalesced polymer region is significantly reduced (see Figure 17). Consequently, we envision the chains in a c-polymer sample to be possibly organized [77] as indicated in Figure 18.

**Figure 19:**
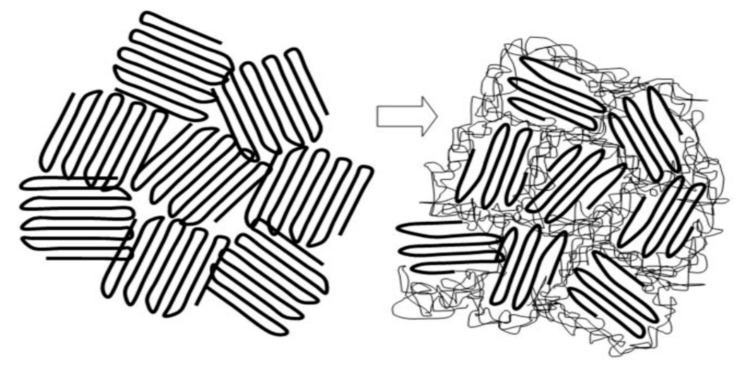
McLeish model for the heterogeneous melt structure produced by the slow heating low-temperature melting of the outer portions of the original single-chain UHMW-PE crystals (**left**) to form entangled normal melt regions. These partition the remaining crystal cores into “cells” (**right**). On further melting, the inner regions of these cells contain un-entangled melt. Figure adapted with permission from Reference [78]. Copyright 2007 The Royal Society of Chemistry Ltd.

More recently, McLeish [78] has suggested “In the case of these heterogeneous melts, however, chains leaving and entering the original ‘cells’ (single-crystal interiors) must result in strong elastic deformations of the entangled material of the cell walls (detached chain-ends). This will result in a free-energy barrier to chain motion…single-chain (non-cooperative) reptation may be strongly suppressed by elastic deformation of the partitioning regions (initially detached and entangled chain-ends, see Figure 19.” Assuming the morphology of polymers initially coalesced from their CD-IC crystals resembles that of the right-hand drawing in Figure 19, but without tie-chains connecting the randomly arranged regions of extended and un-entangled chains, can we (or you) suggest why its transformation to a randomly-coiling entangled melt takes so long?

It seems likely that the chain-ends emerging from each individually coalesced sample region, resulting from the individual polymer-CD-IC crystals from which they were coalesced, should be able to rapidly entangle. Once this occurs, however, it would appear difficult to entangle the central portions of the essentially parallel and extended coalesced chains, as in the case of the heterogeneous melts produced from nascent UHMW-PE single-chain crystalline samples. Then, why does it take so much longer for the melt of an initially coalesced polymer to become fully randomly-coiled and entangled, even though their chains are more than an order of magnitude shorter than those of the UHMW-PEs?

The reason may have its basis in the topological distinction(s) between their initial semi-crystalline and/or the presumed as-coalesced morphologies, as indicated in the right and left hand drawings, respectively, in Figure 18 and Figure 19. As the nascent single-chain UHME-PE crystals melt by detachment from the ends of the single crystals and their chain-ends entangle, the un-entangled central portion of each PE chain remains more mobile, though their reptative motion is restricted by the entanglement of their previously detached chain-ends [75,78]. In addition to a reptation hindered by entanglement of their chain-ends, the un-entangled central chain portions in the coalesced polymer melt are initially more extended and closely packed with a density higher than that of its homogeneous melt. This likely reduces their mobility and may lead to a significant enhancement of the elastic strain placed on the already entangled chain-ends, thereby further retarding their full entanglement. The distinctions between the inter-chain packing densities and mobilities of the initially un-entangled chain portions of coalesced polymers and those of the un-entangled and mobile central portions of UHMW-PE chains individually detached from their single-chain crystals may account for the increased sluggishness of the transition to a fully entangled randomly-coiled melt exhibited by polymers coalesced from their CD-ICs.

## 4. Polarized Optical Examination of Solid and Molten asr- and c-PCL Films

Films of asr-PCL and c-PCL coalesced from its U-IC were examined with a Nikon Eclipse 50i POL polarized optical microscope (POM) (Nikon, Tokyo, Japan) as they were heated from room temperature to 90 °C well above their *T*_m_s (~60 °C) and then cooled to 30 °C at a rate of 10 °C/min using a Mettler FP82HT hot stage (Mettler Toledo, Greifensee, Switzerland) controlled by a Mettler FP90 central processor.

Observations of both the asr- and c-PCL melts above *T*_m_ to 90 °C revealed no differences. No birefringence indicative of polymer chain ordering or anisotropic chain packing were observed. On cooling from their melts at 10 °C/min, the crystalline spherulites formed in the cooled c-PCL film were smaller and uniform in size compared to those in the asr-PCL film which were heterogeneous and larger in size.

Because the POM observations yielded no distinctions between the chain organizations in asr- and c-PCL, we will continue our search for an experimental probe that will be able to make this distinction. The distinct behaviors of these PCL melts must logically have a structural basis.

## 5. Summary and Conclusions

We have presented an up to date summary of our observations of bulk polymer samples that have been reorganized by the formation of and coalescence from their channel structure ICs formed with CD and U hosts. Amorphous polymers coalesced from their CD-ICs (and U-ICs) evidence significantly higher glass-transition temperatures, *T*_g_s. Coalesced semi-crystalline polymers show higher melting and crystallization temperatures, and sometimes different crystalline polymorphs. Samples coalesced from ICs containing two or more guest homopolymers or guest block copolymers exhibit intimate mixing between their common homopolymer chains or between the blocks of the previously included copolymer. Most striking of the unique behaviors evidenced by coalesced polymer samples is the retention of their distinct behaviors even after long-term high temperature melt annealing.

## Figures and Tables

**Table 1:** Measured Densities for as-received and coalesced PVAcs. Adapted with permission from Reference [72]. Copyright Joijode et al. North Carolina State University 2014.

Sample	Density at 25 °C (g/cm^3^) (below *T*_g_)	Density at 58 °C (g/cm^3^) (above *T*_g_)
asr-PVAc	1.093	1.040
c-PVAc (γ-CD)	1.156	1.077
c-PVAc (urea)	1.154	1.076

**Table 2:** % Crystallinity and mechanical properties (mean ± standard error) of asr and c-PCL Fibers. Reprinted (adapted) with permission from Reference [73] Gurarslan, A.; Caydamli, Y.; Shen, J.; Tse, S.; Yetukuri, M.; Tonelli, A.E. *Biomacromolecules* **2015**, *16*, 890–893. Copyright 2015 American Chemical Society.

Physical properties	asr-PCL fiber	c-PCL fiber	Drawn asr-PCL fiber	Drawn c-PCL fiber
modulus (MPa)	41 ± 6	271 ± 20	465 ± 12	770 ± 32
elongation at break (mm)	197 ± 19	110 ± 8	32 ± 2	14 ± 2
% crystallinity	40.6	50.1	50.8	53.3

**Table 3:** Densities of and CO_2_ (0.2 MPa) permeabilities in PET films. Table adapted with permission from Reference [69], Copyright 2013 Wiley Periodicals, Inc.

PET samples	Sample density at 25 °C (g·cm^−3^)	Permeability (P × 10^14^) (cm^3^·s^−1^·Pa^−1^)
asr-PET	1.368	1.64
nuc-PET	1.386	0.57

**Table 4:** *T*_g_s of c-PVAc from its γ-CD-IC annealed above *T*_g_ at 70 °C for different times. Adapted with permission from Reference [72]. Copyright Joijode et al. North Carolina State University 2014.

Annealing time (days)	*T*_g_ (°C)
0	41.5
2	41.7
8	41.5
14	41.2

**Table 5:** Densities of asr-PVAc, c-PVAc (γ-CD), and their 50/50 blend after annealing at 70 °C for 4 weeks ^#^. Adapted with permission from Reference [72]. Copyright Joijode et al. North Carolina State University 2014.

Sample *	Density (g/cm^3^) (above *T*_g_) at 58 °C
asr-PVAc	1.044
c-PVAc	1.077
50/50 = asr/c-PVAc blend	1.076

* *T*_g_ of asr-PVAc film is ~29 °C and for the c-PVAc (γ-CD) and 50/50 blend films *T*_g_s are both ~41 °C. ^#^ The densities of asr- and c-PVAc samples were measured as described in [64] by floatation using water and aq NaBr (21 wt %) (densities of 1.0 and 1.184 g/cm^3^, respectively, lower and higher than that of PVAc). Into a known volume of water, vol (H_2_O), containing a magnetic stirring bar, were placed small pieces of both PVAc films pressed at 70 °C, which sank to the bottom. The NaBr/H_2_O solution was slowly added from a burette, under stirring, until each PVAc film in turn rose from the bottom and was suspended in the aq solution, and the volume of added NaBr/H_2_O, vol(NaBr/H_2_O), was noted. The densities of asr- and c-PVAc films were then obtained at both below and above their glass-transition temperatures **ρ** = $\frac{\left[Vol\left(water\right)x\;\rho \;\left(water\right)+vol\;\left(\frac{NaBr}{water}\right)x\;\rho \;\left(\frac{NaBr}{water}\right)\right]}{Vol\;\left(water\right)+\;vol\;\left(\frac{NaBr}{water}\right)}$.
